# A practical guide to collections‐based research on ecogeographic rules

**DOI:** 10.1002/ece3.10211

**Published:** 2023-06-17

**Authors:** Matan Shelomi, Shai Meiri

**Affiliations:** ^1^ Department of Entomology National Taiwan University Taipei Taiwan; ^2^ School of Zoology & The Steinhardt Museum of Natural History Tel Aviv University Tel Aviv Israel

**Keywords:** Allen's Rule, Bergmann's Rule, biogeography, ecogeography, guidelines, methodology

## Abstract

Ecogeographic research into how species' forms vary across space, time, and climate has taken on new urgency due to contemporary global climate change. Research using museum specimens and other records to study biological rules like Bergmann's, Allen's, and Gloger's Rules has a long history and continues to generate publications and robust scientific debates. Despite the prevalence and history of the field, however, no simple guide on how to carry out such work has ever been published. To lower the barriers of entry for new researchers, this review was created as a practical guide on how to perform ecogeographic research. The guide consolidates disparately published methodologies into a single, convenient document that reviews the history and present of the field of ecogeographic rule research, and describes how to generate appropriate hypotheses, design experiments, gather, and analyze biotic and geographic data, and interpret the results in an ecologically meaningful manner. The result is a semi‐standardized guide that enables scientists at all levels from any institution to carry out an investigation from start to finish on any biological rule, taxon, and location of their choice.

## 1 INTRODUCTION

Body size, coloration, and form are important animal traits, representing a combination of genetic and plastic adaptations to the biotic and abiotic environment. Certain patterns in morphological variation across geographical space and time were noted to hold relatively frequently within certain clades and so were formulated as ecogeographic rules, usually with an ecological explanation that made sufficient sense (Gaston et al., 2008) (Table 1). Some, such as Allen's, Gloger's, and, especially, Bergmann's Rule, have been extensively studied. Considerable debate and controversy exists over most of these rules, such as in which organisms they are meant to apply, whether they are interspecific or intraspecific, what are the underlying factors driving them, how to appropriately research them, when and how to declare a rule as supported, and even what they should be called (Ashton, 2001; Chown & Gaston, 2010; Jones et al., 2005; Mayr, 1956; Meiri, 2011; Meiri et al., 2004; Olalla‐Tárraga, 2011; Pincheira‐Donoso, 2010; Ray, 1960; Rypel, 2014; Watt et al., 2010). Such debates illustrate that the field of ecogeographic studies is vibrant and relevant decades and even centuries since some of these rules were formulated, and anthropogenic climate change has brought new urgency toward the study of how organisms vary with climate (Cohen et al., 2018; Ryding et al., 2021; Santoro & Calzada, 2022; Tian & Benton, 2020; Weeks et al., 2020).

**TABLE 1:** Biological rules involving morphological features, and the explanations for them and original taxa they were formulated for by their authors (though considerable controversy exists for most of these points).

Name	Rule	Explanation	Original taxa	Reference
Allen's Rule	Appendages are disproportionately shorter and thicker in colder climates	Thermoregulation	Endotherms	Allen (1877)
Bergmann's Rule	Populations and species are larger in colder climates	Thermoregulation (food availability, fasting endurance)	Endotherms	Bergmann (1847), Blackburn et al. (1999), James (1970)
Bogert's Rule	Pigmentation is more intense in cold climates	Thermoregulation	Ectotherms	Bogert (1949)
Cope's Rule (Depéret's Rule)	Within lineages, body size increases over evolutionary time	Inherent fitness advantage of large size, predation pressure, food availability, thermoregulation	Terrestrial mammals	Depéret (1907), Rensch (1948)
Deep Sea Gigantism	Organisms are larger in deep water than in shallow water	Temperature, food availability, predation pressure, dissolved O_2_	Invertebrates	Timofeev (2001)
Fasting Endurance Hypothesis 1	Organisms are larger in more seasonal climates	Fasting endurance/starvation resistance	Baleen whales; mammals	Boyce (1979), Brodie (1975)
Fasting Endurance Hypothesis 2	Superorganisms are larger in temperate climates	Fasting endurance/starvation resistance	Social insects	Kaspari and Vargo (1995)
Gloger's Rule	Pigmentation more intense in the tropics	Heat and humidity (UV radiation, latitude, camouflage, evapotranspiration)	Birds and mammals	Gloger (1833)
Harrison's Rule	Host and parasite body sizes covary positively	Food availability, variability, fecundity	Parasites	Harrison (1915)
Hesse's Rule (Heart‐Weight Rule)	Species in cold or high‐altitude environments have larger hearts in relation to body weight	Thermoregulation, hypoxia	Endotherms	Hesse (1924)
The Island Rule (Foster's Rule)	Small organisms show gigantism and large ones show dwarfism on islands compared to mainland relatives	Predation pressure, food availability, competition, optimal body size	Mammals	Foster (1964), Van Valen (1973)
Jordan's Rule	The number of meristic characteristics (originally just vertebrae number) of fish increase in colder waters	Thermoregulation (phyletic position, swimming mode, body size, Lindsey's pleomerism)	Fish	Jordan (1891), Lindsey (1975), McDowall (2008)
Murphy's Rule	Animals will be larger in seasonal environments with a greater temperature range	Fasting endurance, food availability	Palearctic endotherms	Meiri et al. (2005), Murphy (1985)
Temperature‐Size Rule	Ectotherms are larger when reared in colder temperatures (in the laboratory)	Size at maturity, reproductive strategies, development rates	Ectotherms	Atkinson (1995), Ray (1960)

*Note*: Under explanations, modern alternatives to the explanation originally formulated are given in parentheses. This list does not include rules unrelated to morphology.

What follows is a semi‐standardized guide for those who wish to carry out research in this field, especially young investigators beginning their work. It does something not found in most papers written about ecogeographical rules, by delving into issues regarding study design and hypothesis generation, and then describing how to actually analyze museum specimens, find geographic data, perform statistical analysis, and interpret the results in an ecologically meaningful manner. Using this guide, researchers at all levels can design and carry out an investigation into any organism[s], any ecogeographic rule, and any location, from start to finish.

## 2 THEORETICAL UNDERPINNINGS

The most widely studied of the ecogeographic rules, which this guide focuses on, are Bergmann's and Allen's Rules, which respectively state that organisms in colder climates should have larger bodies and disproportionately shorter appendages, meaning a smaller surface‐area‐to‐volume ratio. Latitude or altitude are often used as proxies for climate, as is time in studies examining size changes due to contemporary climate change (Ryding et al., 2021; Teplitsky & Millien, 2014; Tseng et al., 2018) or across geological epochs (Dayan et al., 1991; Hill et al., 2008; Smith et al., 1995). Thus, for example, Bergmann's Rule could be formulated as predicting animals are larger at higher latitudes or are presently shrinking with time. The explanation, as hypothesized by the authors of those rules, is that larger organisms with low surface‐to‐volume ratios can retain heat better in cold climates, while smaller organisms can dissipate heat better in warm climates (James, 1970; Watt et al., 2010). This explanation works well for endotherms (Pincheira‐Donoso, 2010), but less so for small ectotherms (Belk & Houston, 2002; Mousseau, 1997; Partridge & Coyne, 1997; Pincheira‐Donoso, 2010; Pincheira‐Donoso & Meiri, 2013; Ray, 1960; Shelomi, 2012).

Other variables complicate the relationships between temperature, latitude, and size. Organisms with life spans under 1 year may be more strongly affected by seasonal variation. Nocturnal, fossorial, or cave‐dwelling animals or those that avoid climate extremes through hibernation, torpor, migration, etc., will be affected by temperature differently than others (Hantak et al., 2021). Seasonality affects annual minimum, maximum, and mean temperatures differently, and Bergmann‐type clines in a species may change at the latitudes where seasonality becomes apparent (Mousseau & Roff, 1989; Murphy, 1985).

Climate is not necessarily the only, or even main, driver of size. Food availability has been argued by some to be the primary driver of Bergmann‐type size clines (McNab, 2010; Olalla‐Tárraga, 2011; Rosenzweig, 1968; Watt et al., 2010; Yom‐Tov & Geffen, 2006). Net primary productivity (NPP), is occasionally used as an indirect measure of food availability, and has been found to correlate with body size in several studies (Kaspari, 2005; Meiri et al., 2008; Wolverton et al., 2009). It also correlates with climatic indices such as precipitation and is affected by large‐scale climate patterns (Yom‐Tov & Geffen, 2011). Climate can affect food availability in other ways: In Sweden, warming climate was hypothesized to indirectly affect otter (*Lutra lutra*) size by reducing the length of time ice‐covered freshwater lakes and restricted food access (Yom‐Tov, Roos, et al., 2010). Humans can also affect food availability in non‐climatic ways: In Norway, increasing otter size was correlated with increased fish farming (Yom‐Tov, Heggberget, et al., 2006). Urbanization was found to correlate positively with synanthropic mammal size in North America, with the effect attributed to food availability and predator release (Hantak et al., 2021; Yom‐Tov & Geffen, 2011), although not all studies agree (Dubiner & Meiri, 2022).

Other biotic and abiotic interactions could cause sizes to change in ways unrelated to temperature. Examples include competition (Yom‐Tov et al., 1999), predation (Gosler et al., 1995), and distance to food resources (Meiri et al., 2007). Size of introduced stoats (*Mustela erminea*) in New Zealand correlates with the availability of rodent prey, which in turn correlates with the availability of seeds of Southern beech trees (*Notofagus* sp.) that produce their heaviest crops at 3–5 year intervals (Powell & King, 2008). Long‐distance migrating birds show greater temporal size declines in wing chord than short‐distance migrants (Van Buskirk et al., 2010), possibly due to climate change making migrants arrive out of sync with peak food supply (Jonzén et al., 2007). Forest fragmentation due to urbanization can produce terrestrial “islands,” where the Island Rule would come into effect (Fietz & Weis‐Dootz, 2012; Guralnick et al., 2020). These cases exemplify the difficulty in finding a mechanistic explanation for observed size trends. All such variables and more need to be considered when generating hypotheses and designing, conducting, and interpreting studies on ecogeographic rules.

## 3 METHODS FOR EXPERIMENTAL DESIGN

### 3.1 Inter‐ versus intraspecific studies

Much debate exists on whether Bergmann's Rule, for example, applies to interspecific or intraspecific studies, or both (Meiri, 2011; Olalla‐Tárraga, 2011). Increasing availability of large datasets of animal morphology and geographic distributions has led to a proliferation of interspecific, ecogeographic studies (Blackburn & Gaston, 1996; Cardillo, 2002; Olalla‐Tárraga et al., 2006; Reed, 2003; Rodríguez et al., 2006). In such studies, each species is usually assigned a single, mean, or median body size that is regressed on single values of relevant climatic variables (e.g., mean annual temperature, NPP), usually while accounting for phylogenetic non‐independence. Alternatively, a species is assigned to all latitudinal bands or geographic “grid cells” it inhabits, then single, mean, or median values of body size and climatic characteristics are assigned to each species/cell and regressed, usually accounting for spatial non‐independence. These interspecific analyses are statistically sophisticated, powerful, and phylogenetically and geographically comprehensive.

This guide, however, focuses on the intraspecific approach heralded by Rensch (1948) and Mayr (1956). In this approach, one compares the morphologies of individuals or populations within one, widespread species' range. This method can be used to perform an interspecific study by comparing individuals of a genus, family, or other clade rather than a species (Riemer et al., 2018), but such studies may be less likely to report statistically significant relationships than species‐level studies (Shelomi, 2012), as different species within a clade may show different size clines that are obscured when pooling their data. A more appropriate method may be to separately analyze intraspecific trends first, then try to generalize across the species or use meta‐analyses to identify for which organisms under which conditions an ecogeographic rule holds or not. Even for intraspecific studies, some populations benefit from being analyzed separately, such as migrants in native versus non‐native habitats, or the aforementioned Swedish and Norwegian otters.

How many species within a clade must follow a rule to determine whether or not the rule holds for that clade? Mayr (1956) suggested that a rule is only a valid proposal if more than half of the species in a clade adhere to it, which is rather generous (Blackburn et al., 1999). However, one should limit the analysis to species hypothesized to follow the given rule in the first place. For example, if a highly endemic species has a range too small to have significant thermal variance, then the species cannot possibly follow temperature‐related ecogeographic rules, and so is not worth studying or including in such calculations.

### 3.2 Sample size, variable span, and contiguity

Not surprisingly, studies examining a larger number of specimens are more likely to produce significant trends (Yom‐Tov & Geffen, 2011). Adequate statistical power is needed to detect actual effect size, meaning one should ignore tiny sample sizes to avoid false negatives; yet also be wary when interpreting huge samples that can produce statistically significant yet biologically meaningless results.

Examining too small a span or range of predictor variable (number of years, degrees latitude, degrees centigrade, NPP levels, etc.) may result in a narrow range of conditions and thus too weak an effect of those conditions over that range to be statistically detectible. The size cline in an overly small range also may not represent the patterns across the organism's whole range (Shelomi, 2012). A specific example is the gray wolf (*Canis lupus*), which switches from following Bergmann's Rule to following its converse around 60–65° N, forming a hump‐shaped size cline (Geist, 1987). Contiguity of the span must also be considered, as a trend may change across an impassable geographic border (Shelomi, 2012; Shelomi & Zeuss, 2017). Ideally studies should cover contiguous populations, though for some studies the variability among non‐contiguous populations is itself the topic of investigation (e.g., studies of islands or introduced species).

As in geographical ranges, small time ranges may miss important changes. In the 20th century, warming occurred between the 1910s and 1940s and again from the 1970s to the present, yet cooling periods occurred during the 50s and late 70s (Hegerl et al., 2018). A study of Danish stone martens found their skull size correlated with these warming and cooling periods (Yom‐Tov, Leader, et al., 2010). Studies explicitly looking for temporal changes should use data spanning decades or even centuries (Yom‐Tov & Geffen, 2011); however, while longer spans are required to detect glacial/interglacial differences, they may obscure the effects of anthropogenic warming. Even studies not explicitly studying variation over time should cover suitably long timespans, as such variation may nonetheless exist and be important. Note that longer timespans do not necessarily correlate with greater climatic change: A time span from 1750 to 1950 is longer than 1950 to 2020, but it covers less climatic change and habitat destruction, for example.

Seasons can play a large role in the size of individuals, such that monthly effects matter more than effects over years. One example is the Dehnel effect, in which shrews (and potentially other animals) shrink before winter and periods of scarcity (Ochocińska & Taylor, 2003). Researchers may thus want to limit analysis to samples collected in certain months (Maher & Shelomi, 2022). Age is also a factor, especially for long‐lived species and those that show indeterminate growth (Kozłowski, 1996), such as many reptiles. Organism size may depend on seasonal food availability, and so knowing when a specimen was a juvenile probably matters more than when it was collected as an adult. Unfortunately, museum specimens typically are labeled with collection date, not the individual's birth year.

Ultimately, the greatest factor affecting the geographic area and time span one will investigate is practicality. It is easier and cheaper to access specimens in the area where one lives, and museum collections usually have more domestic than international samples. The time span of these collections depends on many factors, such as the age of the museum, the history of the nation, changes in regulations regarding specimen collection from the wild, and the random chance of having researchers interested in creating large collections. Alternative sources for data include field data, bird ringing records, or macroecological data from the literature.

### 3.3 Organism, individual, and parameter

Detecting an existing morphological trend is relatively easy, but identifying the causal mechanism behind the trend is not, as it may be highly specific to the population under investigation. Creativity and background knowledge of the species' local ecology on the part of the investigator is needed, or cooperation with other scientists, which should be factored into choice of subject organism along with practical availability of specimens at museums.

Within a species, one should ideally choose organisms that have stopped growing, meaning adults, though, again, indeterminate growth can be a problem. Likewise, males and females should be treated separately, especially in species that show sexual dimorphism in size (Dayan et al., 1990; Meiri et al., 2004), and sex should be a factor in subsequent analyses. If not accounted for, then random differences in sex ratios across the cline may skew the results. Additionally, male and female sizes may be under different selection regimes (Blanckenhorn et al., 2006; Tarr et al., 2019), showing different size clines affected by different possible drivers (Ciplak et al., 2008; Sepúlveda et al., 2013). Regardless of final analysis, the sex of each specimen should be recorded whenever possible.

When measuring an individual's body size, does one look at body mass, total length, or the length of a certain body part? Again, the answer may be constrained by what is practical. Different preservation methods cause specimens to shrink different amounts (Rosilawati et al., 2014). In pinned insects, head capsules and limbs are usually more stable than abdomens and easier to measure. Body mass allows easy comparisons across organisms of very different shapes, yet it can be highly variable, affected by how recently and how much the individual last ate, whether it is hibernating or migrating, and whether or not it is pregnant or gravid. Furthermore, different body parts may be more or less likely to change size in response to different variables. Wing length is often measured as a proxy for body size in Bergmann's Rule studies in birds (James, 1970), except, as appendages, wings may fall under Allen's Rule, causing contradictory results. Wing length is also under selection pressures that directly relate to age or flight capabilities rather than body size per se (Hamilton, 1961; Merom et al., 1999; Rising & Somers, 1989). Skull dimensions are commonly used for museum specimens of mammals, and skull sutures can enable separation of adults and subadults. In fossil mammals, teeth are often the only well‐preserved element. Teeth also allow for the inclusion of subadults, as teeth erupt in their final size before adult body size is reached. Teeth, however, may be under direct selection for feeding, and thus not correlate well with body size or climate (Dayan et al., 1989, 1991; Dayan & Simberloff, 1994).

## 4 METHODS FOR DATA COLLECTION AND ANALYSIS

### 4.1 Working with museum collections

Young investigators should not feel intimidated when asking to use a museum's specimens. Most natural history museums (and collection managers therein) will be happy to help so long as the sampling is non‐destructive. Contact the person in charge of the taxonomic group of interest and ask how many specimens they have for the relevant species and, if possible, an estimation of their geographic and chronological breadth. This early contact can quickly identify which museums are worth visiting and for how long, and which species have enough specimens for a statistically meaningful study (as inferred with a formal power analysis). Note that the number of specimens in a collection is always larger than what will actually be useful. Some specimens may be lost or on temporary loan, have incomprehensible or missing geographical data, or be juveniles, unsexed, unidentified, broken, or even misidentified. When dealing with older specimens, one will inevitably encounter labels referring to ambiguous locations, or are handwritten and illegible, in a foreign language, or simply missing (Meiri, 2018; Shelomi, 2016). This is all part of the charm of working with museum specimens, and visitors can help the collections by identifying and sometimes rectifying any errors.

Online mapping tools like Google Maps and http://fallingrain.com/world/, plus country‐level resources such as the Gazetteer of Canada, allow fairly accurate conversion of place names to latitude and longitude data that can be matched to climatic data. Some locations are vague (e.g., “India”), so the specimen cannot be pinpointed to a location with enough resolution to match it to the environmental data being analyzed. Best practices guides exist for georeferencing in such scenarios (Chapman & Wieczorek, 2020).

An alternative to directly measuring specimens is remotely analyzing digitized images of specimens from museum websites or depositories such as the Global Biodiversity Information Facility (GBIF.org), VertNet (vertnet.org), or Integrated Digital Biocollections (iDigBio.org) using image analysis software such as ImageJ or PixelZoomer, so long as the images have a scale bar (Merwin et al., 2022). The catch is that most museums have not digitized their entire collection, and so the samples available are few. An intermediate option is analyzing images taken of museum specimens by a collaborating museum staff person. When using records taken from living specimens, such as wing length measurements taken from bird ringing studies, care should be taken to avoid pseudoreplication from the same individual being caught and measured more than once (Yom‐Tov, Yom‐Tov, et al., 2006).

Several papers have noted the existence of “inter‐observer variability,” where different scientists will record different values when measuring the same specimens, so whenever possible the same individual should do all measurements (Tseng & Soleimani Pari, 2019; Yom‐Tov et al., 2013). Otherwise, perform statistical tests to confirm no inter‐observer variability before pooling measurements taken from multiple investigators (Jones et al., 2005).

### 4.2 Sources of bioclimatic and environmental data

While latitude and elevation were once used as proxies for potential predictors like climate, nowadays more direct measurements are readily available online. That said, latitude remains a relatively simple variable for analysis, so long as it is combined with other, thoughtfully chosen variables. Latitude could be used as a continuous variable or analyzed as a categorical value by binning certain ranges of latitude together. An alternative to latitude is two‐dimensional geographic variables, such as cells on a grid, which is also how much environmental data is presented. Sources for gridded, historic bioclimatic data are WorldClim (www.worldclim.org; Fick & Hijmans, 2017) and CHELSA (https://chelsa‐climate.org/; Karger et al., 2017). These GeoTiff files can be analyzed freely with http://app.geotiff.io/. Temperature data can be obtained from weather stations in the vicinity of the geographic points being studied, either directly from the relevant national authorities (Maher & Shelomi, 2022) or resources such as NASA's Surface Temperature Analysis datasets (https://data.giss.nasa.gov/gistemp/station_data_v4_globe/) or the NOAA Global Historical Climatology Network Daily (https://registry.opendata.aws/noaa‐ghcn/). Landsat data from NASA and the US Geological Survey is freely available and can be explored at https://earthexplorer.usgs.gov/, including MODIS Gross and Net Primary Productivity data. A catalog of climate data sources, including paleoclimate, is available at www.realclimate.org/, although some links are broken. At high resolutions, such as single counties or parks, data can be obtained from individual researchers or from specific printed sources.

Other variables may be of interest depending on the organisms studied and hypothesis to be tested. Growing degree days for organisms that require heat above a certain threshold to grow are available from databases such as the USA National Phenology Network or can be calculated from the minimum and maximum annual temperatures as ((*T*_max_ + *T*_min_)/2) − *T*_threshold_. For herbivores, nitrogen content of their hosts is found in the TRY plant trait database (Kattge et al., 2020). For interspecific studies, one should try to test whether phylogenetic relatedness among species correlates with clines (Dubiner & Meiri, 2022; Merwin et al., 2022), which requires corrections using published phylogenies or the necessary molecular data, plus appropriate, “phylogenetic generalized” statistical methods.

### 4.3 Statistical tests

Organizing raw data into a digital dataset or spreadsheet, with dedicated fields containing only numbers for the response and predictor variables and saving backups frequently and before any major data manipulation is essential. Raw datasets should be publicly deposited in a data repository or as supplementary publication data, unless museums have a strict policy against the publication of their raw data, which unfortunately they often have.

Some data may need manipulation such as normalization or smoothing. Log‐transformation of size measurements is common (Tseng & Soleimani Pari, 2019). If body parameters fluctuate during the year, this can be accounted for using a sinusoidal component SIN(2*π* × *m*/12), where *m* is the month numbered in calendar order but with the month with the highest size values as “1” (Yom‐Tov & Yom‐Tov, 2005). Some studies estimated surface area or volume based on length measurements and used that as the dependent variable (Dubiner & Meiri, 2022; Ferreira Amado et al., 2019; Shelomi & Zeuss, 2017), which is valid as Bergmann's and Allen's Rules technically deal with the ratio of surface area to volume, not length per se. Increasingly common is converting the slope of change (e.g., length over latitude) to a percentage change (Hirst et al., 2015), such as proportion of change relative to the mean (Merwin et al., 2022) or through a formula such as [exp^(slope)^ − 1] × 100 (Tseng & Soleimani Pari, 2019), to reduce bias (e.g., a centimeter change is more significant for a 10 cm animal than a 500 cm one). After collecting all the data, if samples are excluded as outliers (Ballinger & Nachman, 2022) or limited according to certain criteria (Maher & Shelomi, 2022), then the rationale for such selections must be reported in the methods section of the study.

A distinct possibility is combining multiple measurements, either for size or the predictor variables, together through tools such as principle component analysis (PCA), typically retaining the first component as a proxy (Juman et al., 2022). This method eliminates intercorrelations, reduces overfitting, and improves visualization, but is harder to interpret and requires standardization. When analyzing PCA data, examine the effects of the independent variables on PC1 by fitting a generalized linear model, and then select the best model using appropriate methods (Brewer et al., 2016; Mac Nally et al., 2018). The interpretation of PCA results needs careful wording: explicitly state which principal component (usually the first) is the proxy and be careful not to interpret the results in terms of the underlying morphological measurements (e.g., just because temperature variables have the highest loading on PC2 does not mean PC2 is “a temperature axis”).

The choice of statistical test depends on the data and hypotheses to be tested. This paper is not a statistics textbook, and authors are advised to collaborate with a statistician or statistically minded biologist if needed. A valid pathway is to start with simple tests such as linear regression or even a non‐parametric rank‐based correlation analysis (especially if the assumption of linearity is violated), and move on to more complex statistics as necessary, for example when non‐linear or additive effects are observed or for highly correlated variables. Try to graph the data first: this will often help infer the appropriate statistical test and can also help identify outliers, erroneous measurements, typos, etc. Consider what a reviewer might object to and err on the side of more robust tests. Tests should ideally be capable of identifying non‐linear trends, especially for studies over large geographic or temporal ranges. As the data will likely be multicollinear (e.g., latitude and precipitation, temperature, seasonality, and altitude), one can first assess variable importance using conditional random forests, one‐way ANOVA, or linear regressions, and then not include redundant or non‐essential variables in the final, multivariable model (Maher & Shelomi, 2022; Merwin et al., 2022). Ultimately a model will need to simultaneously test the effects of several independent values (e.g., time, location, and bioclimatic variables) on the response variable (e.g., size or principal component). Tools for this include multiple regressions, generalized or multiple ANOVA, additive mixed‐effects models, linear mixed‐effects models, general additive models, and others. Familiarity with R is increasingly helpful (Tierney, 2012), though researchers can use whatever statistical software they feel most comfortable with.

Finally, note that a sufficiently robust dataset can often be used to test multiple rules and test geographical and temporal models simultaneously (Juman et al., 2022). Such studies testing multiple ecogeographic hypotheses in conjunction are uncommon now, but are expected to become the norm, so both geographic and temporal data should be incorporated into a model whenever possible if supported by the data.

### 4.4 Interpretation of results and follow‐up studies

As stated above, not every apparent geographic or temporal cline in body form is actually caused by climate, temperature, or food availability. Each species should be examined in light of its own ecology and natural history and its interactions with other organisms and with humans in that particular location. At some point data from multiple populations and species will need to be combined, through generalization or quantitative (and phylogenetically informed) meta‐analyses, to determine whether or not a rule prevails and under what conditions for what organisms (Meiri & Dayan, 2003).

A common issue in interpretations of ecogeographic results is whether observed effects are due to genetic variation, meaning adaptive evolution, or to phenotypic and plastic variation within an individual's life, which is difficult to determine without transplant or common garden experiments (Angilletta Jr & Dunham, 2003; Weaver & Ingram, 1969). For example, if organisms from a high latitude and a low latitude are taken and reared in the same conditions, will the size differences between them decrease (suggesting plasticity) or remain the same (suggesting genetic encoding)? Such studies are hard to do and often impractical. Instead, cases of animals being introduced in areas of different climates have served as natural laboratories, with varying conclusions (Ballinger & Nachman, 2022; Gilchrist & Huey, 2004; Yom‐Tov et al., 1999; Yom‐Tov & Geffen, 2011).

## 5 CONCLUSIONS

Ecogeographic rules are important tools for describing and understanding spatial and temporal patterns in biological traits and drivers of phenotypic evolution. Efforts to validate these rules can reveal much about the biology of different organisms, including predictions on how they will respond to climate change and environmental disturbances. Research on these rules requires thought into how the research question is framed and asked, and a certain level of creativity and background knowledge of the study organism's ecology in order to correctly interpret the resulting data, though practical matters such as specimen availability often play a large role in directing a study. With this guide, the authors hope future generations of researchers will continue to pursue these fruitful lines of inquiry.

## AUTHOR CONTRIBUTIONS

**Matan Shelomi:** Conceptualization (lead); data curation (lead); formal analysis (equal); funding acquisition (lead); project administration (lead); writing – original draft (lead); writing – review and editing (equal). **Shai Meiri:** Data curation (supporting); formal analysis (supporting); writing – original draft (supporting); writing – review and editing (equal).

## CONFLICT OF INTEREST STATEMENT

The authors declare no conflicts of interest.

## Data Availability

Data sharing is not applicable to this article as no datasets were generated or analyzed during the current study.
